# Association between Manganese Exposure through Drinking Water and Infant Mortality in Bangladesh

**DOI:** 10.1289/ehp.10051

**Published:** 2007-03-27

**Authors:** Danella Hafeman, Pam Factor-Litvak, Zhongqi Cheng, Alexander van Geen, Habibul Ahsan

**Affiliations:** 1 Mailman School of Public Health, Columbia University, New York, New York, USA; 2 Lamont-Doherty Earth Observatory, Palisades, New York, USA

**Keywords:** Bangladesh, drinking water, Health Effects of Arsenic Longitudinal Study, heavy metals, infant mortality, manganese

## Abstract

**Background:**

Manganese is a common natural contaminant of groundwater in Bangladesh. In this cross-sectional study we assessed the association between water manganese and all-cause infant mortality in the offspring of female participants in the Health Effects of Arsenic Longitudinal Study Cohort.

**Methods:**

In 2001, drinking water samples were collected, a history of well use was obtained, and a history of birth outcomes was ascertained. To avoid misclassification of exposure, women were included only if they had been drinking from the same well for most of their childbearing years (marriage years – well years ≤ 2). Of a total of 26,002 births (among 6,537 mothers), 3,837 children were born to women with this profile. The current analysis was based on the portion of these infants (*n* = 3,824) with recorded exposure and outcome status, 335 of whom died before reaching 1 year of age.

**Results:**

Infants exposed to water manganese greater than or equal to the 2003 World Health Organization standard of 0.4 mg/L had an elevated mortality risk during the first year of life compared with unexposed infants [odds ratio (OR) = 1.8; 95% confidence interval (CI), 1.2–2.6]. Adjustment for water arsenic, indicators of social class, and other variables did not appreciably alter these results. When the population was restricted to infants born to recently married parents (marriage year 1991 or after), this elevation was more pronounced (OR = 3.4; 95% CI, 1.5–7.9).

**Conclusions:**

These preliminary findings indicate a possible association between manganese exposure and infant mortality. However, given the methodologic limitations of this study, the association needs to be confirmed through future work.

A large percentage of the population in Bangladesh is highly exposed to arsenic and manganese through drinking water. The nationwide British Geological Survey (2001) found that 35% of the collected groundwater samples exceeded the 1993 manganese World Health Organization (WHO) standard of 0.5 mg/L, and 74% of the tested wells had concentrations higher than the Bangladeshi standard of 0.1 mg/L. The levels of manganese are even higher in portions of Araihazar, Bangladesh (the location of the current study), where 80% of the wells were found to exceed the former WHO standard of 0.5 mg/L (Cheng et al. 2004).

The health effects of manganese exposure in humans are not well understood. Although dietary manganese is an essential nutrient, high intakes of manganese through both inhalational exposures and drinking water have been shown to be toxic (Institute of Medicine Food and Nutrition Board 2002). Manganese is best characterized as a neurotoxin; occupational exposures are associated with a characteristic syndrome called manganism, which involves both psychiatric symptoms and Parkinsonian features (Calne et al. 1994; Dobson et al. 2004; Yamada et al. 1986). Exposure through drinking water has been associated with subclinical neurologic effects in Greek adults (Kondakis et al. 1989) and decreased intellectual function in a pediatric population in Araihazar, Bangladesh (Wasserman et al. 2006).

Although the impact of manganese exposures on fetuses and neonates has not been studied extensively in humans, a number of laboratory studies have found that prenatal and postnatal exposure to manganese is associated with embryotoxicity, fetotoxicity, and decreased postnatal growth in rats and mice. Specifically, subcutaneous and intravenous exposure to manganese was associated with both increased resorptions of fetuses and decreased birth weight (Colomina et al. 1996; Sanchez et al. 1993). Although oral and inhalation exposures did not have embryotoxic or fetotoxic effects, high doses through both of these mediums reduced weight gain (Dorman et al. 2000, 2005; Pappas et al. 1997) and decreased survival (Rehnberg et al. 1980) in neonatal rats.

There is also evidence that pregnant women and neonates retain manganese through the oral route to a greater degree than the nonpregnant adult population. The intestinal absorption of manganese is 70% in the neonatal rat, compared with 1–2% in the adult rat (Keen et al. 1994; Mena 1974). The human infant intestine is more developed than that of the neonatal rat, but remains more permeable to macromolecules than the human adult small bowel (Rehnberg et al. 1980). There is also increased manganese retention in neonatal rats and humans, due in part to reduced biliary excretion (Cotzias et al. 1976; Dorner et al. 1989; Keen et al. 1986; Miller et al. 1975). Furthermore, increased manganese levels have been observed in pregnant women, especially during late pregnancy, possibly due to pregnancy-induced anemia which could increase manganese absorption (Spencer 1999). Thus a high manganese concentration in drinking water might have little effect on the adult population, but heavily affect embryos and neonates.

The infant mortality rate is extremely high in Bangladesh; in 2000, it was reported to be 54.0 per 1,000 live births (United Nations Statistical Division 2000). The most common confirmed causes of infant death are acute lower respiratory infections and neonatal tetanus (Baqui et al. 1998). Here, we assess the association between manganese exposure in drinking water and infant mortality; to our knowledge, this is the first study to do so. Births are reconstructed from a reproductive history of women in the Health Effects of Arsenic Longitudinal Study (HEALS) cohort to address this question.

## Materials and Methods

### Study population

The HEALS cohort is a study of 11,749 participants 18–70 years of age living in Araihazar, Bangladesh, of whom 6,707 are women; the primary aim is to assess the health effects of arsenic exposure. All study subjects gave oral informed consent after Bangladeshi field research physicians explained the purpose and procedures of the cohort study. The study and its consenting procedure were approved by the institutional review board of Columbia University and the Ethical Committee of the Bangladesh Medical Research Council.

Details of HEALS methods have been published elsewhere (Ahsan et al. 2006a, 2006b), and only relevant methods are briefly described here. Individuals were recruited to the HEALS cohort and first interviewed in 2000–2001. A questionnaire, which included a reproductive history and a description of past well use, was administered at this time. Of the 6,707 women in the parent cohort, 6,537 reported at least one live birth. Because water samples were collected after outcomes occurred, well switching in the interim could have led to misclassification of exposure. To minimize this problem, the analysis was restricted to women who drank from the same well for almost all of their reproductive lives; this inclusion criterion was operationalized as *a*) married years minus well years ≤ 2, and *b*) age at marriage < 40 years of age. The former criterion (married years minus well years ≤ 2) was chosen to account for the median first birth interval in Bangladesh, which is between 24 and 36 months (Shaikh 2006). A total of 1,632 women met these conditions, which generated a cohort of 3,837 total live births; this was our target population. Data were missing for either exposure (*n* = 9) or outcome (*n* = 4) on 13 maternal–infant pairs, yielding a sample size of 3,824 infants (born to 1,628 women).

### Assessment of water manganese and arsenic exposure

Water samples were collected as part of a pre-cohort survey of all wells in the study region (van Geen et al. 2003). Field sample collection and laboratory analysis procedures have been previously described in detail (Cheng et al. 2004; van Geen et al. 2003). In brief, samples were collected in 60-mL acid-cleaned polyethylene bottles; 1-mL 7N high purity HCl was added for preservation before shipping to the United States (Lamont-Doherty Earth Observatory of Columbia University) for analysis. Manganese concentrations were determined by high resolution inductively coupled plasma mass spectrometry (HR-ICP-MS; Thermo Elemental, Bremen, Germany), with a detection limit of 0.1 μg/L. Water arsenic concentrations were first determined by graphite furnace atomic absorption spectrometry (Hitachi Z-8200; Hitachi, Tokyo, Japan), which has a detection limit of 5 μg/L. For samples with arsenic concentrations at or below this detection limit, we used HR ICP-MS for quantification.

Arsenic levels were determined for all 5,996 tube wells. Manganese concentrations were initially ascertained for a subset of 1,508 wells, for the purposes of other studies nested within the parent cohort. This first batch of analyses was conducted for two distinct purposes. Several of these studies were clinic-based, and assessed particular effects of trace element exposure on children’s intellectual function and neurologic function (Hafeman et al. 2005; Wasserman et al. 2004, 2006). The remaining studies were well-water surveys for testing the accuracy of a field kit and demonstrating that the composition of well water did not vary significantly over time, and selection was well based (van Geen et al. 2004). Of these wells, 492 were used by women in our target population. A total of 1,408 infants were exposed to well water collected for these purposes; 937 were exposed to water analyzed only for well-based studies, whereas 471 used wells selected for clinic-based studies. For the present study, manganese exposure was subsequently determined for the remaining infants who were eligible for inclusion. Specifically, manganese concentration was quantified in a second batch of 807 wells (*n* = 2,416 infants), yielding manganese exposure for a total of 1,299 wells (*n* = 3,824 infants). Both sets of laboratory analyses were conducted on well water samples obtained at baseline (2000–2001).

Well depth was also quantified for all study tube wells. In a very broad sense, concentrations of arsenic and manganese decline with well depth in Bangladesh (British Geological Survey 2001; van Geen et al. 2003). Well depth has also been found to be inversely associated with bacterial contamination (Embrey and Runkle 2006).

### Assessment of outcome

A reproductive history was taken of female participants at baseline, including the number of pregnancies, the number of live births, and the status of each pregnancy (stillbirth, spontaneous abortion, death, and/or still alive). If the child died, the age at death was recorded; otherwise, the current age of the child was entered. Date of birth was not directly ascertained, but can be determined for the children who are still alive (because current age was determined). The cause of death was not recorded.

The primary outcome assessed was infant death, which was defined as the death of a child that occurred ≤ 1 year of age. Children who survived past this age (whether or not they subsequently died) were considered free of the outcome. Stillbirths and spontaneous abortions were excluded from analysis.

### Assessment of covariates

Information about socioeconomic status (SES), height and weight of mother, birth order, sex of child, year of marriage, age of mother, and a well use history was collected at baseline. A detailed food frequency questionnaire was used to assess manganese consumption of the mother. The methodology and reliability of the food frequency questionnaire have been described previously (Chen et al. 2004). Information on housing construction materials (an indicator of SES) was collected at the second follow-up of the cohort, 2–3 years after baseline.

The following covariates were assessed as potential confounders: water arsenic (as collected), well depth (< 50 feet; ≥ 50 feet), maternal age at marriage (< 30 years; ≥ 30 years), marriage year (as collected), maternal education (as collected), TV access (yes/no), land ownership (yes/no), paternal occupation (daily laborer, farmer, factory worker, business owner, other/unemployed), wall type (tin, cement, other), floor type (mud, concrete, other), maternal weight (as collected), child sex, birth order (first vs. later), maternal dietary manganese (as collected), and batch of water analysis (previous studies vs. current study).

### Statistical analysis

Water manganese was first assessed as a dichotomous variable (with a cut-off at the current WHO level of 0.4 mg/L) and according to quintile of exposure. Because observations were nested in both families (range, 1–10 children per mother) and wells (range, 1–23 children per well), this analysis did not meet the statistical assumption of independence. Thus, for all analyses we used generalized estimating equations (GEE), specifying a dichotomous outcome variable and binary distribution, and observations nested within both mother and well. Because we wished to generalize our findings to individual children (and not the clusters to which they belong), informative clustering was not considered to affect our analysis; thus, standard GEE was chosen over cluster-weighted GEE or other similar techniques (Hoffman et al. 2001; Williamson et al. 2003).

We estimated odds ratios (ORs) and 95% confidence intervals (CIs) of the association between manganese and infant mortality using GEE. Four models were fit: *a*) unadjusted for potential confounders; *b*) adjusted only for well variables (water arsenic and depth); *c*) adjusted for indicators of SES (maternal education, family land ownership, paternal occupation, TV access, and housing type); *d*) adjusted for all measured covariates. For individuals missing data on continuous covariates, values were imputed from sample means; missing categories were created for categorical variables with missing data. To address specific study weaknesses, we divided the population into subgroups and fit adjusted models: *a*) firstborn children versus not firstborn children and *b*) recent events (defined as children born to mothers married since the median marriage year) versus nonrecent events.

Data on the concentration of 29 other inorganic constituents of groundwater were available for a subset of the drinking wells (*n* = 173 wells). Individual correlations between manganese and the measured minerals were determined. Using GEE, the OR (95% CIs) between manganese exposure and infant mortality was constructed for this population. The following models were fit in this subset: *a*) unadjusted and *b*) adjusted for each constituent, individually. Joint confounding was not assessed.

## Results

### Study population characteristics

**Figure 1:**
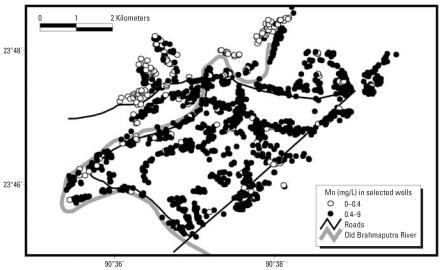
Map of wells with known manganese concentration in the 26-km^2^ study site, according to manganese levels (< 0.4 mg/L vs. ≥ 0.4 mg/L) (*n* = 1,299 wells).

Most (84.5%) of the infants in our study population were exposed, either directly or through maternal intake, to water manganese levels above the WHO standard (0.4 mg/L). The median manganese concentration was 1.28 mg/L, ranging from 0 to 8.61 mg/L. Figure 1 is a map of the wells in the study area, according to manganese concentration (< 0.4 mg/L vs. ≥ 0.4 mg/L). Some geographic clustering of manganese values is evident; however, low and high manganese wells are distributed throughout the 26-km^2^ study area.

Table 1 shows the frequency of covariates, according to exposure status. Manganese concentration is highly correlated with other well characteristics. Infants exposed to high levels of manganese were much more likely to be exposed to elevated arsenic levels (*p* < 0.0001). Well depth was negatively associated with manganese concentration (*p* < 0.0001); most unexposed individuals were drinking from wells with a depth of > 50 feet. Several measures of SES varied according to exposure status. Forty-three percent of the unexposed infants were born to mothers who reported access to a TV, whereas only one-third of exposed infants’ mothers reported such access (*p* = 0.002). Exposed infants were also more likely to live in houses with mud (vs. concrete) floors and tin (vs. cement) walls than their unexposed counterparts (*p* = 0.0002 and 0.01, respectively).

### Association between manganese and infant mortality

Of 3,824 children included in this analysis, 335 died before reaching the age of one year. Table 2 shows the unadjusted association between water manganese concentration and infant mortality, categorized at the WHO standard of 0.4 mg/L (OR = 1.8; 95% CI, 1.2–2.6) and by manganese quintile. No clear dose–response curve was observed. The crude association did not change appreciably with the addition of well characteristics, measured socioeconomic variables, or all measured covariates. Adjustment for batch (prior studies versus the present study) did not alter the association between water manganese concentration and infant death (Table 2). However, batch differences in the main association were observed. Stratifying results according to the purpose of water analysis (clinic-based studies, well-based studies, and present study), we found a relationship between dichotomized exposure and outcome in the clinic-based samples (OR = 2.8; 95% CI, 0.9–8.9) and well-based samples (OR = 2.7; 95% CI, 1.4–5.3), but found no association in the samples analyzed for the purposes of the current study (OR = 1.1; 95% CI, 0.6–1.8).

### Additional analyses

Information on period between births, an important predictor of infant mortality, was not measured. To rule out the possibility that this predictor was confounding the association, we restricted the population to first births (*n* = 1,530). The association observed between manganese (dichotomized at the WHO standard) and infant mortality in the overall sample was similar in this subset of individuals (OR = 2.0; 95% CI, 1.1–3.6) (Table 2).

This study population was constructed specifically to avoid the misclassification of exposure caused by well switching. However, misclassification could still arise if participants failed to provide an accurate well-use or reproductive history. The resultant bias would be expected to have a disproportionate impact on nonrecent events, rendering the recent events more reliable. To look at this hypothesis, we divided the study population at the median of marriage year (as a proxy for birth year). The data indicate that more recently married individuals (within the previous 10 years) gave a more complete reproductive history than those married before 1991. Specifically, the infant mortality rate of children born to those married between 1991 and 2000 was 82 per 1,000 (0.082), which is within the range of statistics given by the United Nations common database for Bangladesh during this period (0.096 in 1990 to 0.054 in 2000) (United Nations Statistics Division 2006). However, the infant mortality rate for those married between 1981 and 1990 (0.094) was less than the expected range (0.129 in 1980 to 0.096 in 1990), and the rate for those married between 1971 and 1980 (0.090) was well below the country-wide statistics for this decade (0.145 in 1970 to 0.129 in 1980). The association between manganese (dichotomized at the WHO standard) and the outcome was stronger in the population born to parents married after 1991 (adjusted OR = 3.4; 95% CI, 1.5–7.9) than in those married earlier (OR = 1.3; 95% CI, 0.8–2.2) (Table 2).

To assess the potentially confounding effects of other constituents of groundwater, we conducted an additional analysis on a subset of our population (*n* = 473 infants drinking from 173 wells) for whom water concentration data were available on 29 metals. In this subset, the unadjusted association between water manganese and infant mortality was higher than in the entire study sample (OR = 3.4; 95% CI, 0.9–13.8). Table 3 shows the correlations with manganese and the impact of adjustment for the most highly correlated minerals (*r* > 0.2). No individual metal explained the entire association between water manganese and infant mortality, although adjustment for water silicon concentration did slightly attenuate the estimate (silicon-adjusted OR = 2.8; 95% CI, 0.7–11.6).

## Discussion

This study suggests an association between manganese exposure and infant mortality in a human population. This effect was not explained by other measured predictors of infant mortality, such as SES, birth order, and child sex. Analysis restricted to first-born children indicates that interpregnancy interval—an important predictor of infant mortality—did not bias these results. Furthermore, the described association is unlikely to be fully explained by the effects of other constituents of drinking water that were analyzed, as demonstrated in the overall and subgroup analysis. Maternal dietary manganese also did not confound these results.

Because outcome occurred before exposure was measured, temporality and misclassification of exposure present the largest problem in this analysis. We addressed this in two ways. First, the reconstructed cohort included only the offspring of individuals who had used the same well for most (i.e., all but 2 years or less) of the years of marriage. Second, we divided the study population at the median year of marriage (proxy for year of birth), and found a larger association in the more recent events (associated with a more reliably classified exposure and outcome status).

The results differed according to the batch of water manganese analysis; although a large effect of exposure was found in the samples analyzed for prior studies, no association was seen in the sample analyzed specifically for this study. The most likely explanation for this discrepancy is that populations selected for prior studies differed systematically from the remaining population. Specifically, there might be an effect modifier of manganese exposure that is present in the selected populations, but not the remaining population. One candidate for such a variable is water arsenic concentration; low arsenic (< 5 μg/L) was a selection criteria for two studies (a well-based and a clinic-based study) in the first batch. Although 42% of the infants in the first batch were exposed to low arsenic, none of the infants in the second batch (for the purposes of the current study) were exposed to levels < 5 μg/L. Within the first batch, a stronger effect was seen in the infants exposed to < 5 ug/L arsenic (OR = 3.7; 95% CI, 1.5–8.8) than those exposed to higher levels (OR = 1.8; 95% CI, 0.8–4.0) (*p*-value for interaction = 0.2). Thus it is possible that manganese has a greater effect on infant mortality in the absence of arsenic exposure; however, this result is based on a post hoc analysis, and further study is necessary for confirmation.

Other less likely explanations for the discrepancy between batches are chance, laboratory error, and selection bias in the first batch. We consider the latter explanation unlikely because an appreciable association between manganese and infant death is observed in both well-based and clinic-based studies. Such a bias (involving differential selection of exposure and outcome) would be unlikely to occur identically in studies with such different selection processes.

Alternative explanations should be considered for this finding. First, it is possible that residual confounding by socioeconomic status biased these results. We measured the covariates at baseline, after the study events had already occurred. It is possible that these variables, as measured at the time of outcome, would indeed explain the association. This is particularly a problem for TV access and maternal weight, which might have changed over time.

Second, it is possible that manganese is correlated with another contaminant in well water, which explains the association. The analysis of 29 inorganic constituents of groundwater in a subset of the population (*n* = 473) addressed this possibility to some extent, and demonstrated that adjustment for individual minerals did not appreciably alter the association. However, this analysis was done only on a subset of the population, and thus might not have been representative. In addition, it is possible that other constituents of groundwater that were not analyzed could potentially play a role. For example, the above analysis does not address the possibility of bacterial contamination. In a cross-sectional survey of Bangladesh, 54% of selected tube wells failed to meet the WHO standard of no detectable fecal coliforms (Hoque 1999). To the extent that bacterial contamination is predicted by well depth, adjustment for the latter variable addressed this alternative explanation. Adjustment for well depth did not appreciably alter the association between manganese exposure and infant mortality, indicating that the observed relationship was not attributed to this third variable. However, an association between manganese and bacterial contamination, independent of well depth, could explain the observed results.

Finally, it is possible that measurement error could explain these findings. These include both misclassification of exposure (due to well switching) and misclassification of outcome (due to misreporting stillbirths or deaths after 1 year of age as infant death). We would expect such misclassification to be nondifferential, which would generally bias results toward the null. However, if measurement error is considerable, differential misclassification has been shown to occur unpredictably (Jurek et al. 2005); in addition, correlated measurement error (even if it is nondifferential) could bias results away from the null (Chavance et al. 1992; Jurek et al. 2005).

A few previous studies have assessed the association between manganese and reproductive outcomes in humans, and the findings have been null. In a nested case–control study (77 cases, 1,177 controls), Aschengrau et al. (1989) found that community water manganese concentration was not associated with the risk of stillbirth. In addition, a birth cohort study (106 mother–infant pairs) found that placental manganese was not associated with birthweight (Osman et al. 2000). The findings of these studies can be easily reconciled with the present results. First, human studies have not looked at the association between manganese and infant mortality. As noted in the introduction, manganese might affect weight gain of neonates, as seen in several animal studies (Dorman et al. 2000, 2005; Pappas et al. 1997; Rehnberg et al. 1980); this would affect infant mortality, but not birth weight or stillbirths. Second, these studies do not have highly exposed participants. In the nested case–control study by Aschengrau et al. (1989), individuals were considered exposed if they drank water manganese ≥ 0.02 mg/L. This is an order of magnitude lower than the WHO recommendation used for this study (≥ 0.4 mg/L).

For water manganese to have the proposed health effects, the fetus or neonate must be directly exposed to the metal. There are three possible routes of exposure that are compatible with our hypotheses: placenta, breast milk, and drinking water. Although placental transfer does occur (Widdowson et al. 1972), only a small percentage of this concentration passes to the fetus (Kontur and Fechter 1985). Thus it is unlikely that placental transfer represents the primary route of exposure. Breast milk is normally very low in manganese (Freeland-Graves 1994), although an elevated concentration of manganese in breast milk has been associated with high levels of inhalational exposure (Sharma and Pervez 2005). However, manganese is not fat-soluble; thus the concentrations in breast milk are still below blood levels (Golding 1997; Sharma and Pervez 2005), and breast milk is not a concentrated source of the metal. Finally, it is possible that neonates are exposed directly through drinking water. Breast-feeding rates are very high in Bangladesh (Arifeen et al. 2001). However, neonates are not regularly fed colostrum, and the initiation of breast-feeding is often delayed; sugar water is frequently given as a replacement (Arifeen et al. 2001; Das and Ahmed 1995; Greiner 1997). Furthermore, exclusive breast-feeding is far from universal; in the Bangladesh Demographic Health Survey (1999–2000), 70% of infants had been given supplementary food by 6 months of age (Giashuddin and Kabir 2004). Thus most infants are given water both during the first day of life and after 6 months of age. Neither precise age at death (in months) nor a history of breast-feeding was collected for this study, so direct exposure to drinking water remains a consideration.

Several limitations of this study should be taken into account when interpreting these results. First, the data were not collected specifically for this analysis, so certain important variables were not recorded. These included cause of infant death, year of birth (for those infants who died), and history of breast-feeding. Second, this analysis was based on a reproductive history that was obtained, in many cases, several years after the exposure and outcome had occurred. Although various strategies have been undertaken to minimize the effect of exposure misclassification and imprecise covariate measurement on these results, these issues still represent potential threats to the validity of this study. Third, the absence of a clear dose–response relationship between manganese exposure and infant death limits the causal inference that can be made based on these findings. Although it is possible that this relationship is truly nonlinear, the appearance of a threshold also makes alternative explanations for this finding more likely.

In conclusion, these results indicate a possible association between water manganese exposure and all-cause infant mortality that should be further explored. Most important, these results should be replicated in an *a priori* designed study with more reliable methods for assessing exposure, outcome, and covariates. In addition, the association between manganese exposure and more specifically defined mortality outcomes (i.e., early neonatal deaths, deaths from acute lower respiratory infection) should be assessed. Finally, potential routes of exposure should be investigated; for instance, the interaction between breast-feeding behavior and water manganese should be evaluated.

## Figures and Tables

**Table 1:** Characteristics of infants born to mothers participating in HEALS (*n* = 3,824), according to exposure status [no. (%)].

Variable	Mn < 0.4 mg/L	Mn ≥ 0.4 mg/L	*p*-Value^a^
Sex			
Male	314 (53)	1,626 (50)	0.86
Female	282 (47)	1,602 (50)	
TV access			
Yes	257 (43)	1,060 (33)	0.002
No	339 (57)	2168 (67)	
Maternal education (years)			
0–4	262 (44)	1,695 (53)	0.22
5–15	334 (56)	1,533 (47)	
Land ownership			
Yes	313 (53)	1,571 (49)	0.88
No	283 (47)	1,657 (51)	
House type: wall			
Tin	436 (73)	2,388 (77)	0.01
Cement	92 (16)	375 (12)	
Other	37 (6)	109 (4)	
Missing	31 (5)	242 (8)	
House type: floor			
Mud	414 (69)	2,469 (77)	0.0002
Concrete	133 (22)	390 (12)	
Other	18 (3)	109 (3)	
Missing	31 (11)	260 (8)	
Paternal occupation			
Daily laborer	32 (5)	186 (6)	0.80
Farmer	60 (20)	246 (8)	
Factory worker	124 (14)	748 (23)	
Business	134 (18)	800 (25)	
Other/unemployed	90 (18)	419 (13)	
Missing	156 (16)	839 (26)	
Maternal weight, at cohort baseline (kg)			
27.0–43.4	267 (45)	1,596 (49)	0.04
43.5–85.0	329 (55)	1,544 (48)	
Missing	0 (0)	88 (3)	
Water arsenic concentration (μg/L)			
< 10	317 (53)	533 (17)	< 0.0001
≥ 10	279 (46)	2,695 (83)	
Well depth (feet)			
23–48	167 (28)	1,683 (52)	< 0.0001
50–240	419 (70)	1,455 (45)	
Missing	10 (2)	90 (3)	

a Clustering of observations within family and well accounted for, using GEE.

**Table 2:** Crude and adjusted ORs (95% CIs) for the association between water manganese concentration and infant mortality.

	Dichotomous (mg/L)		Quintiles (mg/L)				
Models	< 0.4	≥ 0.4	Q1: 0–0.5	Q2: 0.5–1.0	Q3: 1.0–1.6	Q4: 1.6–2.1	Q5: 2.1–8.6
Whole study population (*n* = 3,824)							
Unadjusted	1.0 (ref)	1.8 (1.2–2.6)	1.0 (ref)	1.8 (1.2–2.7)	1.6 (1.1–2.4)	1.3 (0.9–2.0)	2.0 (1.4–3.0)
Adjusted for water arsenic and depth	1.0 (ref)	1.8 (1.2–2.8)	1.0 (ref)	1.9 (1.2–2.9)	1.7 (1.1–2.6)	1.4 (0.9–2.2)	2.1 (1.4–3.2)
Adjusted for indicators of SES^a^	1.0 (ref)	1.7 (1.1–2.5)	1.0 (ref)	1.8 (1.2–2.7)	1.6 (1.0–2.4)	1.3 (0.8–1.8)	1.9 (1.3–2.8)
Adjusted for all measured covariates^b^	1.0 (ref)	1.9 (1.2–2.9)	1.0 (ref)	1.9 (1.3–3.0)	1.8 (1.1–2.9)	1.5 (0.9–2.4)	2.3 (1.5–3.6)
Subsets							
First-born children (*n* = 1,530)^b^	1.0 (ref)	2.0 (1.1–3.6)	1.0 (ref)	2.1 (1.1–3.9)	1.6 (0.8–3.1)	1.9 (0.9–3.8)	2.4 (1.2–4.8)
Not first-born children (*n* = 2,294)^b^	1.0 (ref)	1.8 (1.0–3.1)	1.0 (ref)	1.8 (1.0–3.2)	1.9 (1.1–3.5)	1.0 (0.5–1.9)	2.1 (1.1–3.8)
Married after 1991 (*n* = 1,891)^b^	1.0 (ref)	3.4 (1.5–7.9)	1.0 (ref)	2.5 (1.2–5.3)	2.6 (1.2–5.8)	2.0 (0.9–4.4)	3.3 (1.6–7.1)
Married in or before 1991 (*n* = 1,933)^b^	1.0 (ref)	1.3 (0.8–2.2)	1.0 (ref)	1.6 (0.9–2.7)	1.3 (0.7–2.3)	1.0 (0.5–2.0)	1.5 (0.9–2.8)

ref, referent.

a Maternal education, maternal weight at baseline, land ownership, TV ownership, housing characteristics, and paternal occupation.

b Water arsenic and depth + SES variables + marriage year, marriage age, child sex, birth order, dietary manganese, and batch.

**Table 3:** Association between manganese and infant mortality, adjusted individually for the constituents of groundwater that have the highest correlation with manganese (*r* > 0.2).^a^

Model	Correlation with Mn^b^	< 0.4 vs. ≥ 0.4 mg/L Mn OR (95% CI)
Unadjusted	—	3.4 (0.9–13.8)
Silicon	0.41	2.8 (0.7–11.6)
Lithium	0.36	3.4 (0.8–13.7)
Uranium	0.34	3.6 (0.9–14.5)
Magnesium	0.33	3.6 (0.9–14.1)
Sulfur	0.32	3.9 (0.9–16.0)
Calcium	0.29	3.1 (0.8–12.5)
Sodium	0.22	3.4 (0.8–13.9)
Strontium	0.21	3.1 (0.8–12.3)
Vanadium	–0.34	3.8 (0.8–16.9)
Phosphorus	–0.36	3.9 (1.0–15.3)

*n* = 473 participants, nested in 173 wells.

a Other measured inorganic materials: potassium, iron, aluminum, chromium, cobalt, nickel, copper, zinc, molybdenum, cadmium, indium, tin, antimony, cesium, barium, rhenium, mercury, lead, bismuth.

b All correlations significant at *p* < 0.0001.
